# Vaccination Coverage Among Children Aged 19–35 Months — United States, 2017

**DOI:** 10.15585/mmwr.mm6740a4

**Published:** 2018-10-12

**Authors:** Holly A. Hill, Laurie D. Elam-Evans, David Yankey, James A. Singleton, Yoonjae Kang

**Affiliations:** 1 Immunization Services Division, National Center for Immunization and Respiratory Diseases, CDC.

The Advisory Committee on Immunization Practices (ACIP) recommends routine vaccination by age 24 months against 14 potentially serious illnesses (*1*). CDC used data from the 2017 National Immunization Survey-Child (NIS-Child) to assess vaccination coverage at national, state, territorial, and selected local levels among children aged 19–35 months in the United States. Coverage remained high and stable overall, exceeding 90% for ≥3 doses of poliovirus vaccine, ≥1 dose of measles, mumps, and rubella vaccine (MMR), ≥3 doses of hepatitis B vaccine (HepB), and ≥1 dose of varicella vaccine. Although the proportion of children who received no vaccine doses by age 24 months was low, this proportion increased gradually from 0.9% for children born in 2011 to 1.3% for children born in 2015. Coverage was lower for most vaccines among uninsured children and those insured by Medicaid, compared with those having private health insurance, and for children living outside of metropolitan statistical areas* (MSAs), compared with those living in MSA principal cities. These disparities could be reduced with greater awareness and use of the Vaccines for Children^†^ (VFC) program, eliminating missed opportunities to vaccinate children during visits to health care providers, and minimizing interruptions in health insurance coverage.

The NIS-Child is a random-digit–dialed telephone (cellular and landline) survey of parents/guardians of children aged 19–35 months in the 50 states, the District of Columbia, selected local areas, and U.S. territories.^§^ NIS-Child coverage estimates are based on a provider-reported vaccination history. Interviewers request contact information for all the child’s vaccination providers and permission to contact each provider to obtain vaccination records for that child. All identified providers are mailed an immunization history questionnaire to record dates and types of vaccines administered; data from responding providers are combined to create a synthesized vaccination history for each child. NIS-Child methods, including weighting procedures, have been described.^¶^ In 2017, the overall response rate** to the telephone interview portion of the survey was 26.1%. Adequate provider-reported vaccination data^††^ were available for 53.9% of children with a completed household interview, resulting in a sample size of 15,333 children. T-tests on weighted data were used to evaluate differences in coverage estimates by sociodemographic characteristics; differences were considered statistically significant for p-values <0.05. CDC assessed changes in survey accuracy, estimated components of difference between the 2016 and 2017 NIS-Child estimates, and estimated linear trends in vaccination coverage by month and year of birth using weighted linear regression.^§§^ No evidence for change in survey accuracy from 2016 to 2017 was detected (*2*).

## 2017 Vaccination Coverage

Coverage was >90% for vaccination with ≥3 doses of poliovirus vaccine (92.7%), ≥1 dose of MMR (91.5%), ≥3 doses of HepB (91.4%), and ≥1 dose of varicella vaccine (91.0%) (Table 1). Children were least likely to be up-to-date with ≥2 doses of hepatitis A vaccine (HepA) (59.7%), the combined 7-vaccine series^¶¶^ (70.4%), and rotavirus vaccination (73.2%). Coverage with HepB birth dose was also low (73.6%).

**TABLE 1:** Estimated vaccination coverage among children aged 19–35 months, by selected vaccines and doses — National Immunization Survey-Child, United States, 2013–2017*

Vaccine/Dose	Survey year % (95% CI)				
	2013	2014	2015	2016	2017
**DTaP^†^**					
≥3 doses	94.1 (93.2–95.0)	94.7 (94.0–95.4)	95.0 (94.4–95.5)	93.7 (92.8–94.5)^§^	94.0 (93.3–94.7)
≥4 doses	83.1 (81.8–84.3)	84.2 (83.0–85.4)	84.6 (83.5–85.7)	83.4 (82.1–84.6)	83.2 (82.0–84.3)
**Poliovirus (≥3 doses)**	92.7 (91.6–93.6)	93.3 (92.5–94.1)	93.7 (93.0–94.3)	91.9 (90.9–92.9)^§^	92.7 (91.9–93.5)
**MMR (≥1 dose)^¶^**	91.9 (90.9–92.7)	91.5 (90.6–92.4)	91.9 (91.0–92.7)	91.1 (90.1–92.0)	91.5 (90.6–92.3)
**Hib**					
Primary series**	93.7 (92.7–94.5)	93.3 (92.5–94.1)	94.3 (93.7–94.9)	92.8 (91.8–93.6)^§^	92.8 (91.9–93.6)
Full series**	82.0 (80.7–83.3)	82.0 (80.7–83.2)	82.7 (81.5–83.8)	81.8 (80.5–83.0)	80.7 (79.4–82.0)
**HepB**					
≥3 doses	90.8 (89.7–91.7)	91.6 (90.7–92.4)	92.6 (91.9–93.3)	90.5 (89.3–91.5)^§^	91.4 (90.5–92.3)
Birth dose^††^	74.2 (72.8–75.7)^§^	72.4 (70.9–73.9)	72.4 (71.0–73.7)	71.1 (69.5–72.7)	73.6 (72.0–75.2)^§^
**Varicella (≥1 dose)^¶^**	91.2 (90.2–92.1)	91.0 (90.1–91.9)	91.8 (91.0–92.5)	90.6 (89.6–91.5)	91.0 (90.1–91.8)
**PCV**					
≥3 doses	92.4 (91.4–93.3)	92.6 (91.8–93.4)	93.3 (92.5–94.0)	91.8 (90.8–92.7)^§^	91.9 (90.9–92.8)
≥4 doses	82.0 (80.6–83.3)	82.9 (81.6–84.2)	84.1 (83.0–85.2)	81.8 (80.4–83.1)^§^	82.4 (81.1–83.6)
**HepA**					
≥1 dose	83.1 (81.9–84.3)^§^	85.1 (84.0–86.2)^§^	85.8 (84.7–86.8)	86.1 (84.9–87.2)	86.0 (84.8–87.1)
≥2 doses^§§^	54.7 (53.1–56.3)	57.5 (55.9–59.1)^§^	59.6 (58.1–61.0)	60.6 (59.1–62.2)	59.7 (58.2–61.3)
**Rotavirus^¶¶^**	72.6 (71.1–74.0)^§^	71.7 (70.1–73.2)	73.2 (71.8–74.6)	74.1 (72.6–75.5)	73.2 (71.6–74.7)
Combined 7-vaccine series***	70.4 (68.8–71.9)	71.6 (70.2–73.1)	72.2 (70.9–73.6)	70.7 (69.2–72.2)	70.4 (68.9–71.9)
**No vaccinations**	0.7 (0.5–1.1)	0.8 (0.6–1.0)	0.8 (0.6–1.0)	0.8 (0.6–1.0)	1.1 (0.9–1.4)^§^

**Abbreviations:** CI = confidence interval; DTaP = diphtheria, tetanus toxoids, and acellular pertussis vaccine; HepA = hepatitis A vaccine; HepB = hepatitis B vaccine; Hib = *Haemophilus influenzae* type b conjugate vaccine; MMR = measles, mumps, and rubella vaccine; PCV = pneumococcal conjugate vaccine.

* For 2013, children born during January 2010–May 2012; for 2014, children born during January 2011–May 2013; for 2015, children born during January 2012–May 2014; for 2016, children born during January 2013–May 2015; and for 2017, children born during January 2014–May 2016.

^†^ Includes children who might have been vaccinated with diphtheria and tetanus toxoids vaccine or diphtheria, tetanus toxoids, and pertussis vaccine.

^§^ Statistically significant (p<0.05) change in coverage compared with previous survey year.

^¶^ Includes children who might have been vaccinated with measles, mumps, rubella, and varicella vaccine.

** Hib primary series: ≥2 or ≥3 doses, depending on product type received; full series includes primary series and booster dose, which includes receipt of ≥3 or ≥4 doses, depending on product type received.

^††^ One dose of HepB administered from birth through age 3 days.

^§§^ Estimates of ≥2 doses of HepA are likely underestimates because a child could be on schedule but not receive a second dose of HepA until age 41 months. This dose would not be collected by NIS-Child, which includes children aged 19–35 months only.

^¶¶^ Includes ≥2 doses of Rotarix monovalent rotavirus vaccine (RV1), or ≥3 doses of RotaTeq pentavalent rotavirus vaccine (RV5). The maximum age for the final rotavirus dose is 8 months, 0 days.

*** The combined 7-vaccine series (4:3:1:3*:3:1:4) includes ≥4 doses of DTaP, ≥3 doses of poliovirus vaccine, ≥1 dose of measles-containing vaccine, the full series of Hib (≥3 or ≥4 doses, depending on product type), ≥3 doses of HepB, ≥1 dose of varicella vaccine, and ≥4 doses of PCV.

## Vaccination Coverage by Selected Characteristics

Coverage was lower (range = 2.6–6.9 percentage points) for children living in non-MSAs than among those living in MSA principal cities for most vaccines (Table 2). Children living in non-MSAs had a higher prevalence of having received no vaccinations (1.9%) compared with children in MSA principal cities (1.0%).

**TABLE 2:** Estimated vaccination coverage among children aged 19–35 months, by selected vaccines and doses, metropolitan statistical area (MSA) status,* and health insurance status^†^ — National Immunization Survey-Child, United States, 2017^§^

Vaccine/Dose	MSA status % (95% CI)			Health insurance status % (95% CI)			
	MSA, principal city (referent) (n = 6,689)	MSA, non-principal city (n = 5,846)	Non-MSA (n = 2,798)	Private only (referent) (n = 8,536)	Any Medicaid (n = 5,714)	Other insurance (n = 644)	Uninsured (n = 439)
**DTaP^¶^**							
≥3 doses	94.6 (93.4–95.6)	94.1 (92.9–95.0)	91.6 (89.1–93.6)**	96.5 (95.7–97.2)	92.6 (91.2–93.8)**	93.7 (90.7–95.8)**	78.2 (71.3–83.8)**
≥4 doses	85.0 (83.3–86.5)	82.6 (80.6–84.5)	78.1 (74.9–80.9)**	86.9 (85.2–88.5)	80.8 (78.9–82.5)**	83.6 (79.3–87.2)	62.4 (55.0–69.1)**
**Poliovirus (≥3 doses)**	93.2 (91.9–94.4)	92.9 (91.7–93.9)	90.1 (87.4–92.2)**	95.2 (94.3–96.0)	91.2 (89.6–92.5)**	92.7 (89.5–95.0)	77.9 (71.0–83.6)**
**MMR^††^ (≥1 dose)**	92.5 (91.2–93.6)	90.9 (89.3–92.3)	89.9 (88.0–91.6)**	93.7 (92.3–94.8)	90.4 (89.1–91.6)**	91.0 (87.5–93.6)	74.6 (67.5–80.6)**
**Hib**							
Primary series^§§^	93.4 (92.2–94.5)	92.6 (91.1–93.9)	91.2 (88.7–93.2)	95.5 (94.6–96.2)	91.1 (89.5–92.5)**	92.2 (88.8–94.7)**	78.0 (71.1–83.7)**
Full series^§§^	81.6 (79.6–83.4)	80.7 (78.6–82.7)	77.3 (74.1–80.2)**	85.1 (83.2–86.9)	77.7 (75.6–79.7)**	78.8 (73.8–83.1)**	62.0 (54.6–68.9)**
**HepB**							
≥3 doses	92.6 (91.3–93.7)	90.4 (88.7–91.9)**	90.7 (88.8–92.3)	93.3 (91.9–94.4)	90.4 (88.8–91.7)**	92.5 (89.4–94.7)	78.6 (71.8–84.1)**
Birth dose^¶¶^	73.6 (71.1–76.0)	72.8 (70.3–75.1)	76.6 (73.6–79.3)	73.0 (70.9–75.0)	74.7 (72.0–77.2)	71.8 (66.2–76.8)	68.7 (61.9–74.8)
**Varicella^††^ (≥1 dose)**	92.3 (91.0–93.4)	90.4 (88.7–91.8)	88.3 (86.2–90.1)**	92.9 (91.5–94.1)	90.4 (89.1–91.6)**	91.3 (88.0–93.8)	69.5 (62.2–76.0)**
**PCV**							
≥3 doses	92.2 (90.5–93.6)	91.9 (90.4–93.2)	90.6 (88.0–92.6)	94.5 (92.9–95.7)	90.5 (88.9–91.8)**	91.0 (87.6–93.5)**	75.2 (67.9–81.2)**
≥4 doses	83.6 (81.7–85.4)	82.0 (79.9–84.0)	79.1 (75.9–81.9)**	87.6 (85.8–89.3)	78.9 (76.8–80.8)**	81.3 (76.8–85.2)**	59.0 (51.6–66.1)**
**HepA**							
≥1 dose	87.2 (85.3–88.9)	85.7 (83.9–87.4)	82.5 (80.1–84.6)**	88.1 (86.5–89.6)	85.3 (83.5–87.0)**	86.1 (81.7–89.5)	63.3 (55.7–70.3)**
≥2 doses	61.1 (58.7–63.4)	59.2 (56.7–61.6)	56.5 (53.3–59.7)**	63.2 (61.0–65.2)	57.7 (55.2–60.2)**	61.1 (55.2–66.7)	35.7 (29.1–42.9)**
Rotavirus***	73.8 (71.3–76.2)	73.3 (70.7–75.7)	70.5 (67.3–73.6)	81.8 (79.8–83.6)	66.8 (64.2–69.4)**	67.4 (61.0–73.3)**	51.5 (44.2–58.7)**
**Combined 7-vaccine series^†††^**	71.9 (69.7–74.1)	69.8 (67.4–72.2)	66.8 (63.6–69.9)**	76.0 (73.9–77.9)	66.5 (64.1–68.9)**	69.2 (63.6–74.2)**	48.5 (41.2–55.8)**
**No vaccinations**	1.0 (0.7–1.3)	1.1 (0.8–1.5)	1.9 (1.3–2.7)**	0.8 (0.6–1.1)	1.0 (0.7–1.4)	—^§§§^	7.1 (4.6–10.8)**

**Abbreviations**: CI = confidence interval; DTaP = diphtheria and tetanus toxoids and acellular pertussis vaccine; HepA = hepatitis A vaccine; HepB = hepatitis B vaccine; Hib = *Haemophilus influenzae* type b conjugate vaccine; MMR = measles, mumps, and rubella vaccine; PCV = pneumococcal conjugate vaccine.

* MSA status was determined on the basis of household-reported county and city of residence and was grouped into three categories: MSA principal city, MSA nonprincipal city, and non-MSA. MSA and principal city were as defined by the U.S. Census Bureau (https://www.census.gov/geo/reference/gtc/gtc_cbsa.html). Non-MSA areas include urban populations not located within an MSA as well as completely rural areas.

^†^ Children’s health insurance status was reported by parent or guardian. “Other insurance” includes the Children’s Health Insurance Program, military insurance, coverage via the Indian Health Service, and any other type of health insurance not mentioned elsewhere.

^§^ Children in the 2017 National Immunization Survey-Child were born during January 2014–May 2016.

^¶^ Includes children who might have been vaccinated with diphtheria and tetanus toxoids vaccine or diphtheria, tetanus toxoids, and pertussis vaccine.

** Statistically significant (p<0.05) difference compared with the referent group.

^††^ Includes children who might have been vaccinated with measles, mumps, rubella, and varicella vaccine.

^§§^ Hib primary series: ≥2 or ≥3 doses, depending on product type received; full series includes primary series and booster dose, which includes receipt of ≥3 or ≥4 doses, depending on product type received.

^¶¶^ One dose of HepB administered from birth through age 3 days.

*** Includes ≥2 or ≥3 doses, depending on product type received (≥2 doses for Rotarix [RV1] or ≥3 doses for RotaTeq [RV5]).

^†††^ The combined 7-vaccine series (4:3:1:3*:3:1:4) includes ≥4 doses of DTaP, ≥3 doses of poliovirus vaccine, ≥1 dose of measles-containing vaccine, the full series of Hib (≥3 or ≥4 doses, depending on product type of vaccine), ≥3 doses of HepB, ≥1 dose of varicella, and ≥4 doses of PCV.

^§§§^ Estimate not available because the 95% CI was ≥20.

Coverage among children insured by Medicaid was lower (2.5–15.0 percentage points, depending on vaccine) than that among those with private insurance for all vaccines assessed except the HepB birth dose (Table 2). The same pattern was observed among uninsured children: coverage was substantially lower (14.7–30.3 percentage points) than that among those privately insured. Prevalence of uninsured children in the 2017 NIS-Child was 2.8%. This lower vaccination coverage among the uninsured, Medicaid-insured, and those living outside of MSAs was especially evident for diphtheria and tetanus toxoids and acellular pertussis vaccine (DTaP), the full series of *Haemophilius influenzae* type b conjugate vaccine (Hib), and pneumococcal conjugate vaccine (PCV), that require a booster dose in the second year of life. In addition, the proportion of uninsured children who had received no vaccinations (7.1%) was higher than that among those with private insurance (0.8%). The proportion of unvaccinated children was similar among children insured by Medicaid and those with private insurance. Among unvaccinated children in the 2017 NIS-Child, 17.2% were uninsured.

Differences in vaccination coverage by race/ethnicity and poverty status in 2017 were similar to those observed in previous years (Supplementary Table 1, https://stacks.cdc.gov/view/cdc/59414) (*3*). Vaccination coverage also varied by state (Supplementary Table 2, https://stacks.cdc.gov/view/cdc/59415). For example, estimated rotavirus coverage ranged from 64.7% in California to 85.1% in Rhode Island. Coverage with MMR ranged from 85.8% in Missouri to 98.3% in Massachusetts; MMR coverage was <90% for 11 states in 2017.

## Trends in Vaccination Coverage

Coverage by month and year of birth remained stable during January 2012–January 2016 for most vaccines (Figure) (*2*). Coverage by age 2 years over 12 consecutive birth months declined by 0.5 percentage points for ≥3 HepB doses and increased by 1.1 percentage points for ≥2 HepA doses (*2*). Coverage with ≥2 HepA doses was higher by age 35 months than by age 24 months (e.g., 75.3% versus 39.6% for children born January 2012) (*2*).

**FIGURE:**
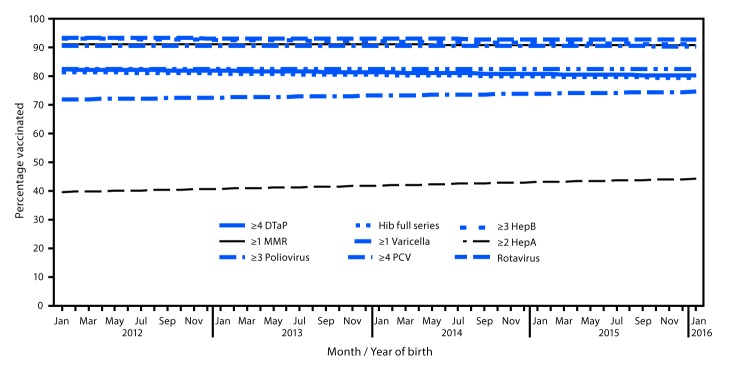
Estimated linear trend in coverage with selected vaccines* by age 24 months,^†^ by month and year of birth^§^ — National Immunization Survey-Child, United States, 2013–2017 **Abbreviations:** CI = confidence interval; DTaP = diphtheria, tetanus toxoids, and acellular pertussis vaccine; HepA = hepatitis A vaccine; HepB = hepatitis B vaccine; Hib = *Haemophilus influenzae* type b conjugate vaccine; MMR = measles, mumps, and rubella vaccine; PCV = pneumococcal conjugate vaccine. * Hib full series: ≥3 or ≥4 doses, depending on product type received (primary series and booster dose). Rotavirus: ≥2 or ≥3 doses, depending on product type received (≥2 doses for Rotarix [RV1] or ≥3 doses for RotaTeq [RV5]). ^†^ Except for rotavirus, vaccination coverage was assessed before the child reached his/her 24-month birthday. The Kaplan-Meier method was used to account for censoring vaccination status for children assessed before age 24 months. Rotavirus vaccination was assessed before the child reached his/her 8-month birthday. ^§^ Estimated linear relationship between month and year of birth and vaccination coverage, based on weighted linear regression analysis using the inverse of the estimated variance of each point estimate to construct the weights. Estimated percentage point change over 12 birth months: ≥4 DTaP −0.55 (95% CI = -1.20 to 0.10); ≥3 poliovirus -0.17 (-0.52 to 0.18); ≥1 MMR -0.11 (-0.58 to 0.35); Hib full series -0.51 (-1.13 to 0.11); ≥3 HepB -0.53 (-0.97 to -0.09); ≥1 varicella -0.05 (-0.53 to 0.42); ≥4 PCV 0.0 (-0.69 to 0.68); ≥2 HepA 1.13 (0.30 to 1.97); rotavirus 0.68 (-0.09 to 1.45).

HepB birth dose coverage was higher in 2017 (73.6%) than in 2016 (71.1%) (Table 1). Analysis of trends in HepB birth dose coverage by month and year of birth during January 2012–May 2016 indicated no change in coverage, although an increasing trend was estimated for more recent births (January 2014–May 2016) (*2*). The percentage of unvaccinated children increased from 0.8% in 2016 to 1.1% in 2017. By annual birth cohort, the percentage of children with no vaccinations by age 2 years increased from 0.9% for children born in 2011 to 1.3% (47,700 children) for those born in 2015 (Supplementary Figure, https://stacks.cdc.gov/view/cdc/59413), representing an additional 18,400 unvaccinated children.

## Discussion

Overall vaccination coverage among young children remained high and stable in the United States in 2017. However, the findings from this survey highlight several opportunities for improvement. Coverage was lower for most vaccines among uninsured and Medicaid-insured children and among children living outside of MSAs. These disparities were larger for vaccines that require a booster dose in the second year of life (e.g., DTaP, Hib, and PCV). Although the number of children who have received no vaccinations by age 24 months has been gradually increasing, most children are still routinely vaccinated. Continued evaluation of prevalence and reasons for nonvaccination is needed, as are improvements in access to and delivery of age-appropriate vaccinations to all children. CDC continues to examine barriers to early childhood vaccination, including assessing obstacles to and parents’ experiences with accessing vaccination services.

Vaccination coverage differences by insurance status are concerning, given that children insured by Medicaid and uninsured children are eligible for the VFC program, which was designed to remove financial barriers by providing free vaccines to program participants. However, other issues, such as unfamiliarity with the VFC program and how to access it, transportation, child care, and convenience of clinic hours might also need to be addressed if the goals of this important element of the immunization safety net are to be fully realized. Lack of geographic proximity to vaccination providers, including those who participate in the VFC program, can be a barrier to vaccination. The shortage of health care providers, especially pediatricians, might partially explain the lower coverage among children living in rural areas (*4*).

Vaccination coverage could be increased and sociodemographic and geographic disparities reduced with increased administration of all recommended vaccines during provider visits. A study of potentially achievable coverage estimated that 90% coverage would have been attained many years ago for the recommended number of doses of DTaP, PCV, and Hib for children aged 19–35 months if missed opportunities for administration of the final doses of these vaccines had been eliminated (*5*). Reducing missed opportunities would promote timely receipt of all recommended vaccine doses and decrease the amount of time that children remain vulnerable to vaccine-preventable diseases.

The percentage of children who have received no vaccines has increased, reaching 1.3% for children born in 2015, compared with 0.3% among those 19–35 months when surveyed in 2001 (*6*). Some children might be unvaccinated because of choices made by parents, whereas for others, lack of access to health care or health insurance might be factors. Unvaccinated children in the 2017 NIS-Child were disproportionately uninsured: 17.2% of unvaccinated children were uninsured, compared with 2.8% of all children. Evidence-informed strategies addressing parents’ decisions about vaccinating their children could focus on both programs and individual patients, such as vaccine delivery through school programs, strong recommendations by providers to parents to vaccinate their children, and reinforcement of the importance of community protection through vaccination (*7*).

Variation in coverage by health insurance and MSA status and the increasing percentage of unvaccinated children raise concerns about possible pockets of susceptibility in which children are not as well protected as national coverage estimates might indicate. Measles was declared eliminated from the United States in 2000, yet outbreaks caused by imported cases continue to occur each year; 118 measles cases were reported in 2017 (https://www.cdc.gov/measles/cases-outbreaks.html) (*8*). The continued occurrence of measles outbreaks in the United States underscores the need to ensure high MMR coverage among all young children.

The findings in this report are subject to at least two limitations. First, low response rates and lack of access to phoneless households could result in selection bias, which might persist even with application of survey weights designed to minimize such bias. Second, vaccination histories might be incomplete if not all providers were identified or some of those identified chose not to participate. Bias in vaccination coverage estimates has been evaluated in a sensitivity analysis accounting for these potential errors, with results indicating underestimation of actual vaccination coverage by 4 to 5 percentage points (*9*).

Vaccination coverage among young children could be improved through higher participation by both children and providers in the Vaccines for Children program. Consistent access to health insurance is another important element of the immunization safety net. Barriers to participation in the VFC program should be identified and eliminated so that all eligible children have the opportunity to access recommended vaccines. A number of evidence-based strategies have also been described that could enhance these efforts to increase vaccination coverage, such as notifying parents when children are due for a vaccination, establishing standing orders or policies that allow nonphysician personnel to administer vaccines, and enhancing computerized immunization information systems for tracking vaccinations (https://www.thecommunityguide.org/topic/vaccination) (*10*). Continued vaccination coverage assessment using the NIS-Child can guide efforts to improve vaccination coverage and protect children from vaccine-preventable diseases and better understand the low but increasing prevalence of nonvaccination.

**Summary**

**What is already known about this topic?**

The Advisory Committee on Immunization Practices recommends routine vaccination by age 24 months against 14 potentially serious illnesses.

**What is added by this report?**

In 2017, coverage with most recommended vaccines among children aged 19–35 months remained stable and high but was lower in more rural areas and among uninsured or Medicaid-insured children. A small but increasing proportion of children received no vaccines by age 24 months.

**What are the implications for public health practice?**

Collaboration with state immunization programs, eliminating missed immunization opportunities, and minimizing interruptions in insurance coverage are important to understand and address coverage disparities among children eligible for the Vaccines for Children program and those in rural areas.

## Footnotes

* MSA status was determined on the basis of household-reported city and county of residence and was grouped into three categories: MSA principal city, MSA nonprincipal city, and non-MSA. MSAs and principal cities were as defined by the U.S. Census Bureau (https://www.census.gov/geo/reference/gtc/gtc_cbsa.html). Non-MSA areas include urban populations not located within an MSA as well as completely rural areas.

† https://www.cdc.gov/vaccines/programs/vfc/index.html.

§ Estimates for states, selected local areas, and the territory of Guam are available online (https://www.cdc.gov/vaccines/imz-managers/coverage/childvaxview/data-reports/index.html). The local areas sampled separately for the 2017 NIS included areas that receive federal Section 317 immunization funds and are included in the NIS sample every year (Chicago, Illinois; New York, New York; Philadelphia County, Pennsylvania; Bexar County, Texas; and Houston, Texas) and three additional sample areas (El Paso County, Texas; Dallas County, Texas; and Travis County, Texas). The 2017 NIS-Child was also conducted in Guam, Puerto Rico, and U.S. Virgin Islands; however, data collection in Puerto Rico and U.S. Virgin Islands was suspended because of the severity of the 2017 hurricane season, resulting in insufficient data for estimation of vaccination coverage. National estimates in this report exclude all territories.

¶ Details regarding the statistical methodology of NIS-Child are available in the NIS-Child Data User’s Guide 2016. https://www.cdc.gov/vaccines/imz-managers/nis/datasets.html.

** The Council of American Survey Research Organizations (CASRO) household response rate is calculated as the product of the resolution rate (percentage of the total telephone numbers called that were classified as nonworking, nonresidential, or residential), screening completion rate (percentage of known households that were successfully screened for the presence of age-eligible children), and the interview completion rate (percentage of households with one or more age-eligible children that completed the household survey). The CASRO household response rate is equivalent to the American Association for Public Opinion Research type 3 response rate. http://www.aapor.org/AAPOR_Main/media/publications/Standard-Definitions20169theditionfinal.pdf.

†† Children with at least one vaccination reported by a provider and those who had received no vaccinations were considered to have adequate provider data.

§§ https://www.cdc.gov/vaccines/imz-managers/coverage/childvaxview/pubs-presentations/NIS-vax-trends-2012-2016.html.

¶¶ The combined 7-vaccine series (4:3:1:3*:3:1:4) includes ≥4 doses of DTaP; ≥3 doses of poliovirus vaccine; ≥1 dose of measles-containing vaccine; ≥3 or ≥4 doses (depending upon product type) of Hib; ≥3 doses of Hep-B; ≥1 dose of varicella vaccine; and ≥4 doses of PCV.
